# The SARM1 TIR NADase: Mechanistic Similarities to Bacterial Phage Defense and Toxin-Antitoxin Systems

**DOI:** 10.3389/fimmu.2021.752898

**Published:** 2021-09-23

**Authors:** Aaron DiAntonio, Jeffrey Milbrandt, Matthew D. Figley

**Affiliations:** ^1^ Department of Developmental Biology, Washington University School of Medicine in Saint Louis, St. Louis, MO, United States; ^2^ Needleman Center for Neurometabolism and Axonal Therapeutics, Washington University School of Medicine in Saint Louis, St. Louis, MO, United States; ^3^ Department of Genetics, Washington University School of Medicine in Saint Louis, St. Louis, MO, United States

**Keywords:** NAD^+^, innate immunity, NMNAT2, axon degeneration, plant, metabolism, TIR domain, abortive infection

## Abstract

The Toll/interleukin-1 receptor (TIR) domain is the signature signalling motif of innate immunity, with essential roles in innate immune signalling in bacteria, plants, and animals. TIR domains canonically function as scaffolds, with stimulus-dependent multimerization generating binding sites for signalling molecules such as kinases and ligases that activate downstream immune mechanisms. Recent studies have dramatically expanded our understanding of the TIR domain, demonstrating that the primordial function of the TIR domain is to metabolize NAD^+^. Mammalian SARM1, the central executioner of pathological axon degeneration, is the founding member of the TIR-domain class of NAD^+^ hydrolases. This unexpected NADase activity of TIR domains is evolutionarily conserved, with archaeal, bacterial, and plant TIR domains all sharing this catalytic function. Moreover, this enzymatic activity is essential for the innate immune function of these proteins. These evolutionary relationships suggest a link between SARM1 and ancient self-defense mechanisms that has only been strengthened by the recent discovery of the SARM1 activation mechanism which, we will argue, is strikingly similar to bacterial toxin-antitoxin systems. In this brief review we will describe the regulation and function of SARM1 in programmed axon self-destruction, and highlight the parallels between the SARM1 axon degeneration pathway and bacterial innate immune mechanisms.

## Introduction

Injured or diseased axons initiate a self-destruction program known as Wallerian degeneration. SARM1 triggers this pathological axon degeneration (1), and is a key driver of pathology in models of chemotherapy-induced peripheral neuropathy (2–5), traumatic brain injury (6–10), glaucoma (11), and retinal degeneration (12, 13). SARM1 also participates in antiviral defense. SARM1 triggers axon degeneration following rabies infection (14), presumably to halt the spread of the virus as it travels retrogradely down the axon to the neuronal cell body, and induces neuronal cell death in response to bunyavirus infection (15), killing infected cells and thereby reducing viral spread. Hence, the role of SARM1 in pathological axon degeneration is likely closely linked to its function in antiviral innate immunity.

**Figure 1:**
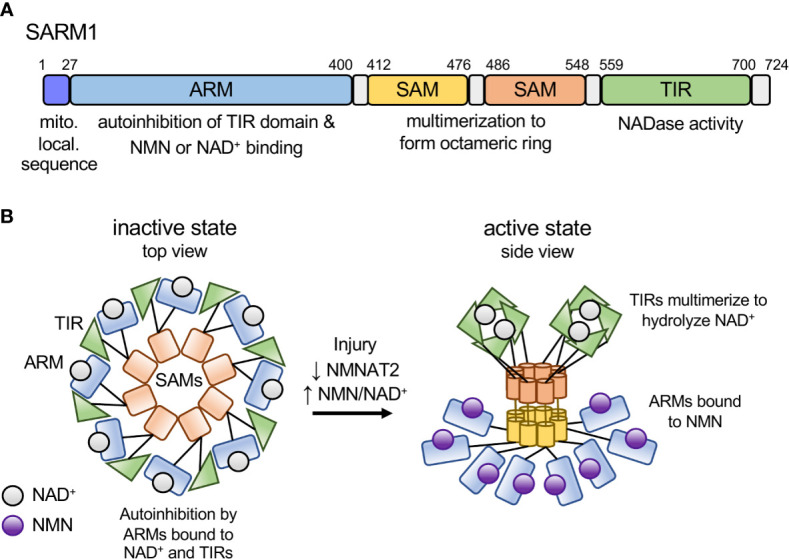
Model of SARM1 domain structure and activation mechanism. **(A)** Domain structure of the human SARM1 protein. SARM1 contains an N-terminal mitochondrial localization sequence and Armadillo-repeat containing domain (ARM), two tandem sterile alpha motif (SAM) domains, and a C-terminal Toll/interleukin-1 receptor (TIR) domain. Numbers denote the amino acid position of the domain boundaries. **(B)** Schematic depicting the activation mechanism of SARM1. In the inactive state SARM1’s ARM domains are bound to NAD^+^ at the allosteric site and bound to adjacent TIR domains both intra- and inter-molecularly, mediating autoinhibition of the TIR’s NADase activity. In response to an increase in the NMN/NAD^+^ ratio, NMN binds to the ARM domain allosteric site, resulting in a conformational change in the ARM domain, disengagement of the ARM-TIR interactions, multimerization of the TIR domains and NADase activity. Based on recent structural data from the RPP1 TIR domain, we depict active TIRs as a tetramer forming two active sites for NAD^+^ binding (20).

SARM1 is a multi-domain protein comprised of an autoinhibitory ARM domain, tandem SAM domains mediating multimerization, and a C-terminal TIR domain NAD^+^ hydrolase (16, 17). In healthy neurons, SARM1 autoinhibition is maintained by multiple intra- and intermolecular interactions (18), including binding of the N-terminal ARM domain to the C-terminal TIR domain (19) (**Figure 1A**). SARM1 autoinhibition is regulated by an allosteric binding site within the autoinhibitory ARM domain that can bind either nicotinamide adenine dinucleotide (NAD^+^) (21, 22) or its precursor, nicotinamide mononucleotide (NMN) (23). NMN promotes SARM1-dependent axon degeneration (24–27). Axon injury leads to loss of the NAD^+^ biosynthetic enzyme NMNAT2 (28), resulting in an increased NMN/NAD^+^ ratio that promotes NMN binding to the allosteric site (23). The switch from NAD^+^ to NMN binding alters the conformation of the autoinhibitory ARM domain, thereby promoting TIR-TIR interactions and enzymatic activity (23) (**Figure 1B**). Below we will highlight commonalities between SARM1 activation and effector mechanisms with similar bacterial innate immune mechanisms.

### SARM1-NMNAT2 Is a Candidate Mammalian Toxin-Antitoxin Pair

**Figure 2:**
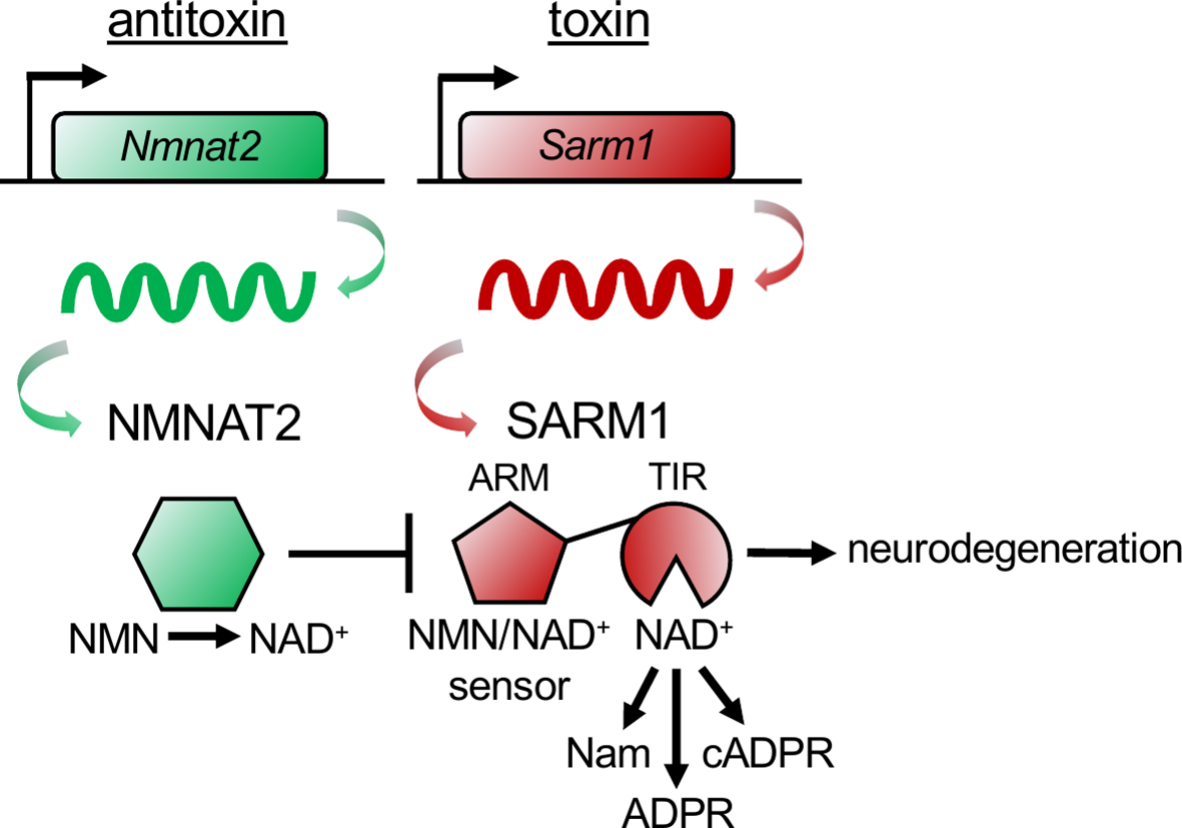
The NMNAT2 antitoxin inhibits the SARM1 toxin to prevent axon degeneration. The antitoxin NMNAT2 converts nicotinamide mononucleotide (NMN) to nicotinamide adenine dinucleotide (NAD+) and thus maintains a healthy NMN/NAD+ ratio in axons. SARM1’s ARM domain senses the ratio of NMN/NAD+ by binding to either metabolite. When the antitoxin NMNAT2 is lost, the NMN/NAD+ ratio rises, NMN binds to the toxin SARM1’s ARM domain, activating SARM1’s TIR domains to hydrolyze NAD+, producing nicotinamide (Nam) and adenosine diphosphate ribose (ADPR), or cyclizing ADPR into cyclic ADPR (cADPR). Activation of the toxin SARM1 drives pathological axon degeneration.

Just as SARM1 can trigger axon self-destruction in response to rabies infection, so too can a bacterial population acquire immunity to bacteriophage infection through an altruistic suicide mechanism known as abortive infection. When infected, the bacterial cells activate a toxin-antitoxin (TA) system prior to phage replication, killing the infected cells and thereby protecting the community by preventing further phage expansion (29). In a TA system, bacteria express both a lethal toxin and its antagonist, the antitoxin. Upon infection, the antitoxin is degraded, unleashing the degenerative activity of the toxin. Genetic deletion of the toxin-encoding gene yields no phenotype in the absence of the inciting stimuli, whereas deletion of the antitoxin-encoding gene results in cell death due to unchecked toxin activity. This lethality can be rescued by concurrent deletion of the toxin-encoding gene. This TA relationship is strikingly reminiscent of the relationship between NMNAT2 and SARM1, with NMNAT2 serving as the antitoxin and SARM1 as the toxin (**Figure 2**). First, NMNAT2 inhibits the prodegenerative activity of SARM1, as antitoxins inhibit the functions of toxins. Second, the classic TA genetic relationship holds for SARM1 and NMNAT2. Loss of SARM1 has no obvious phenotype in mice until an appropriate stimulus, such as neuronal injury, occurs (17, 30). By contrast, genetic loss of NMNAT2 (the antitoxin) is embryonic lethal in mice (31, 32). Double mutants lacking both NMNAT2 and SARM1 fully rescue this lethality (33). Hence, the essential function of NMNAT2 is to inhibit SARM1. Third, similar to a type II TA system in bacteria (29), the antitoxin NMNAT2 is highly labile (28) and the levels of NMNAT2 are a key determinant of SARM1 activation (34). However, in contrast to the bacterial type II TA system, in which the antitoxin inactivates the toxin through direct binding, NMNAT2 inhibits SARM1 indirectly, by modulating the flow of metabolites that bind and regulate SARM1 activity.

To our knowledge, all known TA systems are found in bacteria. Here we posit that *via* convergent evolution SARM1 and NMNAT2 have developed into an analogous TA system to control axonal fate in mammalian neurons. In an injured or unhealthy axon the transport of NMNAT2 is disrupted (3, 28), and neuronal stress pathways promote NMNAT2 turnover (34, 35), leading to the loss of the labile NMNAT2 antitoxin and the subsequent activation of the toxin SARM1, resulting in rapid and efficient axonal self-destruction. This may be a physiological function of SARM1, enabling the phagocytosis and clearance of damaged axons before their contents leak and potentially harm adjacent axons or cells or induce inflammation. Indeed, this scenario was recently demonstrated in a mouse model of ulcerative colitis, in which SARM1 promotes axon degeneration in the enteric nervous system and thereby limits inflammation in the colon (36). It will be interesting to determine whether NMNAT2 is also lost in this colitis model, or in response to rabies infection, when SARM1 is activated and axons degenerate as an antiviral defense (14).

### SARM1 Is the Founding Member of the TIR Domain Family of Innate Immune NADases

The role of SARM1 and its connection to ancient surveillance mechanisms extends beyond its TA relationship with NMNAT2 to its mechanism of degeneration, NAD^+^ cleavage. In many bacterial TA relationships, the toxins are, like SARM1, NAD^+^ glycohydrolases (37). Examples include the toxins SPN (*S. pyogenes*), TNT and MbcT (*M. tuberculosis*), Tne2 (*P. protegens*), and RES (*P. luminescens*), all of which deplete NAD^+^ to induce cellular dysfunction or death and are neutralized by their respective antitoxins (38–42). The newly discovered bacterial TIR domain NADases also participate in phage defense as they are essential components of the Thoeris phage defense mechanism (43, 44). Moreover, bacteria not only use TIR NADases to defend against phage invasion, but also to disrupt mammalian innate immune mechanisms *via* metabolic disruption of the host cell (45). In addition, plant TIR domain innate immune receptors are active NADases and this enzymatic function is essential for the cell death that confers disease resistance (20, 46–48). The conservation of TIR NADase function (**Table 1**) in bacterial, plant, and animal response to infection suggests that TIR-mediated NAD^+^ cleavage is a primordial innate immune function.

**Table 1:** TIR domain-containing proteins with demonstrated intrinsic NADase activity.

TIR domain protein	Organism	References
SARM1	*H. sapiens*	Essuman et al., *Neuron*, (16); Zhao et al., *iScience*, (27); Horsefield et al., *Science*, (47)
SARM1	*M. musculus*	Essuman et al., *Neuron*, (16)
SARM1	*D. rerio*	Essuman et al., *Neuron*, (16)
dSarm	*D. melanogaster*	Essuman et al., *Neuron*, (16)
TirS	*S. aureus*	Essuman et al., *Curr Biol*, (49)
AbTir	*A. baumannii*	Essuman et al., *Curr Biol*, (49)
TcpC	*E. coli*	Essuman et al., *Curr Biol*, (49)
BtpA	*Brucella*	Essuman et al., *Curr Biol*, (49); Coronas-Serna et al., *PLoS Pathog*, (45)
BtpB	*Brucella*	Coronas-Serna et al., *PLoS Pathog*, (45)
PdTir	*P. denitrificans*	Essuman et al., *Curr Biol*, (49)
TcpF	*E. faecalis*	Essuman et al., *Curr Biol*, (49)
ApTir	*Actinoplanes sp.*	Essuman et al., *Curr Biol*, (49)
TcpA	*T. archaeon*	Essuman et al., *Curr Biol*, (49)
TcpO	*M. olleaye*	Essuman et al., *Curr Biol*, (49); Wan et al., *Science* (46)
RBA1	*A. thaliana*	Wan et al., *Science* (46)
RPS4	*A. thaliana*	Wan et al., *Science* (46); Horsefield et al., *Science*, (47)
RPP1	*A. thaliana*	Wan et al., *Science* (46); Horsefield et al., *Science*, (47); Ma et al., *Science*, (20)
BdTIR	*B. distachyon*	Wan et al., *Science* (46); Ofir et al., *bioRxiv*, (44)
L6	*L. usitatissimum*	Horsefield et al., *Science*, (47)
RUN1	*M. rotundifolia*	Horsefield et al., *Science*, (47)
tir-1	*C. elegans*	Horsefield et al., *Science*, (47)
SNC1	*A. thaliana*	Horsefield et al., *Science*, (47)
ROQ1	*N. benthamiana*	Horsefield et al., *Science*, (47)
RPV1	*M. rotundifolia*	Horsefield et al., *Science*, (47)
*Sf*STING	*S. faecium*	Morehouse et al., *Nature*, (50)
ThsB	*B. cereus*	Ofir et al., *bioRxiv*, (44)
ThsB TIR1/TIR2	*B. dafuensis*	Ofir et al., *bioRxiv*, (44)

Finally, SARM1 and evolutionarily diverse TIR domain proteins not only share NADase function, but can also possess regulatory domains controlled *via* allosteric binding to cellular metabolites. The SARM1 TIR domain NADase is fused to a metabolic sensing ARM domain that acts to inhibit the NADase activity until specific environmental signals are present. This is likely a general regulatory mechanism for TIR NADase activation, as organisms from all kingdoms of life encode proteins with TIR domains fused to a variety of other motifs, such as leucine-rich repeat (LRR), tetratricopeptide repeat (TPR), WD repeat, and coiled coil (CC) domains (51–53), that may function as environmental sensors to tune the NADase activity of the fused effector TIR domains. Indeed, this precise regulatory relationship occurs in ancient STING (stimulator of interferon genes) receptor proteins. In both prokaryotes and lower eukaryotes, STING domains are fused with TIR NADase domains. This effectively couples STING domain sensing of cyclic dinucleotides produced by the cyclic AMP-GMP synthase (cGAS) cellular surveillance system to TIR domain NADase activity (50). We suggest that additional multi-domain proteins encoding TIR NADases are likely regulated *via* metabolite binding, sensing changes in cellular metabolism and responding *via* NAD^+^ hydrolysis.

## Discussion

This brief survey of SARM1 and the family of TIR domain NADases demonstrates that mechanistic insights into SARM1 regulation and function have enabled major breakthroughs in our understanding of TIR domain proteins across the domains of life. Key insights from these studies are the identification of SARM1/NMNAT2 as the first candidate mammalian toxin/antitoxin pair, the recognition of multidomain TIR containing proteins as coordinated metabolic sensors and effectors, and the realization that there is a striking commonality between mechanisms of neurodegeneration and the primordial battle between bacteria and bacteriophages.

## Author Contributions

Writing – original draft, all authors. Writing – review and editing, all authors. Funding acquisition, AD and JM. Supervision, AD and JM. All authors contributed to the article and approved the submitted version.

## Funding

This work was funded by National Institutes of Health grants R37NS065053 to AD and RO1NS087632 to AD and JM.

## Conflict of Interest

AD and JM are co-founders, scientific advisory board members, and shareholders of Disarm Therapeutics, Inc. (a wholly-owned subsidiary of Eli Lilly and Company). The editor declared a past co-authorship with all the authors.

The remaining author declares that the research was conducted in the absence of any commercial or financial relationships that could be construed as a potential conflict of interest.

## Publisher’s Note

All claims expressed in this article are solely those of the authors and do not necessarily represent those of their affiliated organizations, or those of the publisher, the editors and the reviewers. Any product that may be evaluated in this article, or claim that may be made by its manufacturer, is not guaranteed or endorsed by the publisher.
